# Paclitaxel induces trained immunity via the GPR183–STING axis to enhance host defense against MRSA infection

**DOI:** 10.1186/s13567-025-01704-8

**Published:** 2026-01-16

**Authors:** Cheng-kai Zhou, Jia-bao Zhang, Yong-jun Yang, Wei Chen, Zhen-zhen Liu

**Affiliations:** 1 Department of Preventive Veterinary Medicine, College of Veterinary Medicine, Jilin University, Changchun, 130062 China; 2 State Key Laboratory for Diagnosis and Treatment of Severe Zoonotic Infectious Diseases, Key Laboratory for Zoonosis Research of the Ministry of Education, Institute of Zoonosis, and College of Veterinary Medicine, Jilin University, Changchun, 130062 China; 3 School of Public Health, Shandong First Medical University and Shandong Academy of Medical Sciences, Jinan, 250000 China; 4 State Key Laboratory of Rare Earth Resource Utilization, Changchun Institute of Applied Chemistry, Chinese Academy of Sciences, Changchun, 130022 China

**Keywords:** Paclitaxel, macrophages, trained immunity, MRSA, infection prevention

## Abstract

**Abstract:**

Methicillin-resistant *Staphylococcus aureus* (MRSA) remains a major global health threat with limited prophylactic options. Trained immunity, characterized by nonspecific functional reprogramming of innate immune cells, offers a promising strategy for infection control. Here, we identify paclitaxel (PTX), a microtubule-stabilizing agent widely used in cancer therapy, as a novel inducer of trained immunity in macrophages. Unlike the microtubule-destabilizing agent nocodazole (Noco), PTX enhanced macrophage proinflammatory responses, phagocytosis, and bacterial killing upon secondary stimulation. Mechanistically, PTX-induced training activated the stimulator of interferon genes protein (STING) pathway, evidenced by increased phosphorylation of STING, TBK1, and IRF3. STING deficiency abolished the trained immune responses and antimicrobial functions. PTX also triggered metabolic reprogramming toward aerobic glycolysis via the Akt–mTOR–HIF1α pathway, which was essential for the trained phenotype. Transcriptomic and functional analyses further revealed that the GPR183–STING axis mediated PTX-induced trained immunity. Inhibition of GPR183 impaired STING activation and suppressed functional responses in vitro. In a murine MRSA pneumonia model, PTX-trained mice showed reduced bacterial burden, preserved lung barrier integrity, and enhanced immune activation, all of which were reversed by GPR183 inhibition or STING deficiency. Collectively, our findings uncover a previously unrecognized immunomodulatory function of PTX and highlight the therapeutic potential of targeting the GPR183–STING axis to enhance trained immunity against resistant bacterial infections.

**Graphical Abstract:**

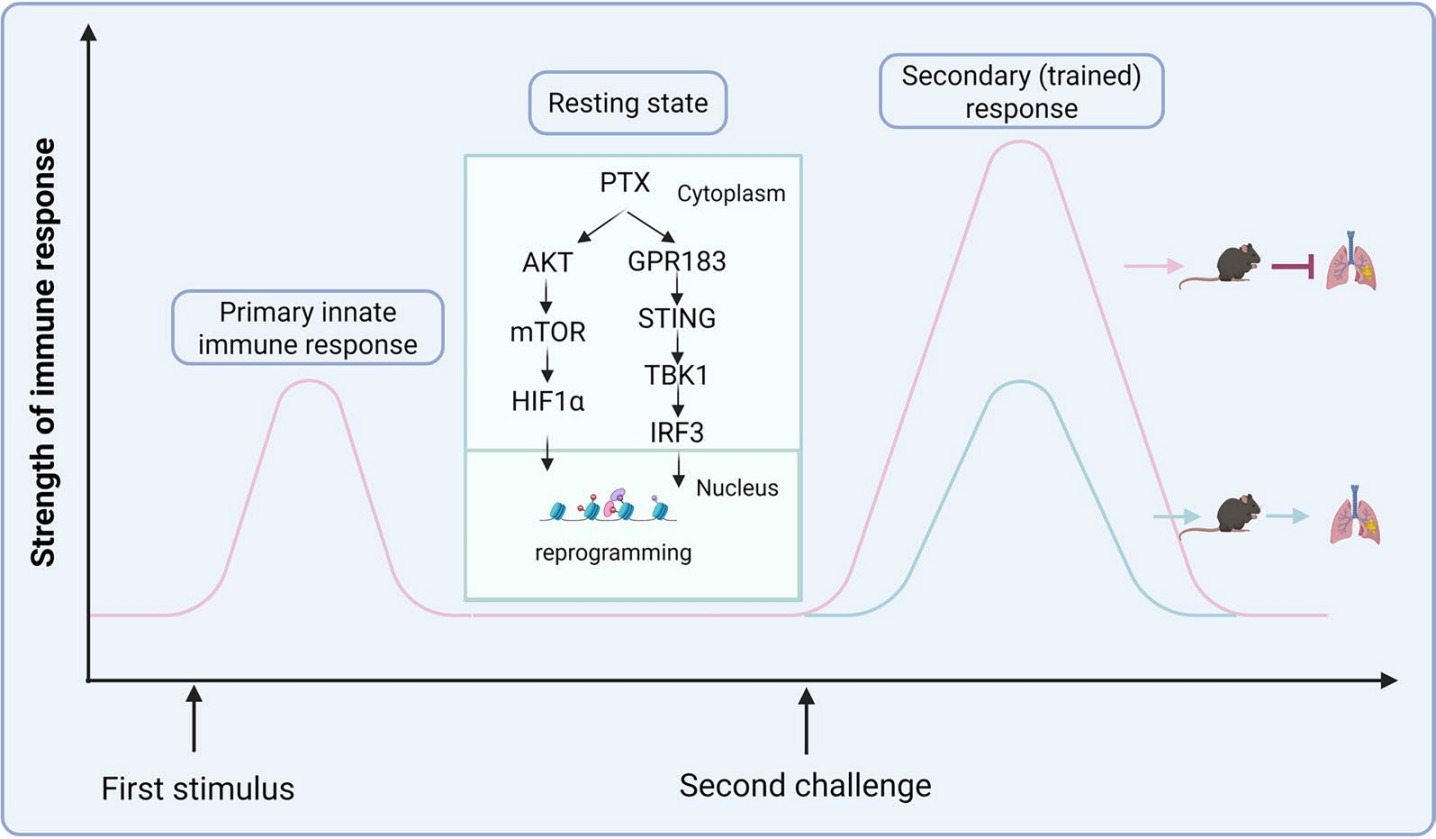

**Supplementary Information:**

The online version contains supplementary material available at 10.1186/s13567-025-01704-8.

## Introduction

Methicillin-resistant *Staphylococcus aureus* (MRSA) maintains its position in the 2024 World Health Organization (WHO) bacterial priority pathogens list (WHO BPPL) high-priority pathogen category, attributing to its serious multidrug resistance and high estimated burden [1]. MRSA causes serious infections in both humans and animals worldwide, leading to enteric infections, post-operative wound infections, sepsis, and pneumonia, which severely impact public health and livestock industry development [2, 3]. The Global Burden of Disease study reported that six pathogens caused more than 250 000 deaths each related to antimicrobial resistance (AMR). The top three pathogens responsible for the deaths attributable to AMR were *E. coli*, *S. aureus*, and *K. pneumoniae*, respectively [4]. To tackle the challenge imposed by MRSA, the development of comprehensive, targeted strategies is necessary including stewardship programs, global surveillance, and enhanced infection prevention and control [5].

Trained immunity, also known as innate immune memory, provides a novel perspective and breakthrough for infection prevention and control [6]. Unlike adaptive immunity which relies on the somatic gene rearrangement of antigen receptors, trained immunity is achieved depending on the epigenetic and metabolic reprogramming after transient stimulation. Specifically, AKT–mTOR–HIF1α-mediated aerobic glycolysis is an important marker of trained immunity [7]. Glutaminolysis, cholesterol metabolism, and fatty acid synthesis are also involved. Certain metabolites derived from these processes, such as acetyl-CoA and fumarate, can control the function of enzymes that modify chromatin, leading to specific histone methylation and acetylation modification (such as H3K4me3 and H3K27ac) of genes involved in the secondary immune responses [8]. The memory property strengthens the immune response to a wide variety of stimuli [9]. On the one hand, trained immunity facilitates stronger innate immune responses to infections and vaccination, providing beneficial heterologous protection [10–12]. Conversely, trained immunity also contributes to the pathophysiology of cardiometabolic, neurodegenerative, autoinflammatory, and allergic diseases [13–16]. The protective effect of trained immunity was demonstrated in different MRSA infectious models, indicating that trained immunity may be considered as an effective control strategy to fight MRSA infection [17, 18]. Searching for trained immunity inducers from plant-derived natural compounds will be a highly promising strategy.

PTX, a well-known chemotherapeutic agent extracted from the bark of the Pacific yew tree (*Taxus brevifolia*), is commonly utilized in treating different types of cancer. Its primary mechanism of action involves stabilizing microtubules, thus interfering with the formation of the mitotic spindle and preventing cell division [19]. However, the immunomodulatory effects of PTX have been largely overlooked in clinical settings, despite their potential implications for prognosis, particularly in tumor recurrence and surgical site infections (SSIs). Trained immunity has been reported as a preventive measure for SSIs, including those resulting from MRSA, the leading cause of SSIs [20]. This investigation was conducted with the aim of investigating the previously unrecognized immunoregulatory functions of PTX in trained immunity. We showed that PTX contributed to trained immunity via the GPR183–STING axis and enhanced host defense against MRSA pneumonia. This study could help establish effective strategies for preventing and controlling MRSA infection and provide a reference basis for clinical rational drug use.

## Materials and methods

### Mice

C57BL/6J wild-type (WT) mice were purchased from Liaoning Changsheng Biotechnology Co., Ltd. Stimulator of interferon genes protein-deficient mice (STING^−/−^) were procured from Jackson Laboratory (Jackson Laboratory, Bar Harbor, USA). Both male and female mice aged 6–8 weeks were used in this study. All experiments involving animals received approval from Jilin University’s Animal Welfare and Research Ethics Committee (SY202511037).

### Trained immunity in vitro

For peritoneal macrophages (PMs), the trained immunity assay was conducted according to the previous methods [21]. Briefly, the differentiated PMs were stimulated with RPMI-1640 medium (31800-022, Gibco, Rockville, USA), β-glucan (5 μg/mL, G5011, Sigma-Aldrich, Saint Louis, USA), PTX (Sigma-Aldrich, 580555), or Noco (Sigma-Aldrich, 487928) for 24 h. β-glucan is a classic inducer of trained immunity [7]. Then, cells were washed and rested for 5 days. On day 6, the PMs were washed and restimulated with lipopolysaccharide (LPS; 100 ng/mL, Sigma-Aldrich, L6529), R848 (100 ng/mL, Sigma-Aldrich, SML0196), Pam3CSK4 (100 ng/mL, tlrl-pms, InvivoGen, San Diego, USA), or *S. aureus* strain USA300 (TCH1516, multiplicity of infection (MOI) = 5 or 50). In the inhibitor experiments, cells were initially treated with MK-2206 (10 μM, S1078, Selleck, Houston, USA), Rapamycin (100 nM, Selleck, S1039), Ascorbate (10 mM, A800295, MACKLIN, Shanghai, China), 2-DG (10 mM, Sigma-Aldrich, D6134), or NIBR189 (10 μM, MACKLIN, N881871) for 24 h, followed by the stimulation with PTX (10 μM).

### Trained immunity in vivo

Mice received intraperitoneal training of paclitaxel (PTX) at low, medium, or high doses (PTX-L, PTX-M, and PTX-H; 5, 10, and 20 mg/kg, respectively). After 7 days, mice were intranasally infected with 1 × 10^8^ colony-forming units (CFUs) of *S. aureus*. The infected serum, bronchoalveolar lavage fluid (BALF), and lung tissues were obtained for quantification of bacterial burden at 24 h post-infection. Besides, the lung tissues were collected for hematoxylin and eosin (H&E) staining. Lung pathology scores were determined according to a previous report [22]. For the inhibitor experiments, NIBR189 was administered to the mice 24 h prior to PTX (20 mg/kg) stimulation.

### Phagocytosis and killing assays

Peritoneal macrophages (PMs) were exposed to *S. aureus* with an MOI of 5. Infection was synchronized by centrifugation at 515 × *g* for 2 min. After 1 h incubation, antibiotics (100 µg/mL gentamicin) were added to kill the remaining extracellular bacteria. The numbers of phagocytosed and intracellular bacteria were assessed after 2 or 4 h of incubation. Percent killing was calculated as (phagocytosed bacteria 2 h − intracellular bacteria 4 h)/ phagocytosed bacteria 2 h.

For immunofluorescence analysis, *S. aureus* was labeled with fluorescein isothiocyanate (FITC) as described previously [23]. Briefly, a suspension of 1 × 10^8^ CFU of bacteria was prepared in phosphate buffered saline (PBS) with 200 μg/mL FITC (ST2065, Beyotime, Shanghai, China). After 30 min of incubation, protected from light, the unbound FITC was removed by washing. Infection was as described above. At the indicated time points, cells were fixed by 4% paraformaldehyde (PFA). After the final washes with PBS, the sections were sealed with a quench-proof tablet containing Hoechst 33342. Pictures were taken with confocal microscopy (Olympus, Tokyo, Japan).

### RNA extraction and quantitative real-time PCR

Total RNA was isolated using RNAiso Plus (9108, Takara, Dalian, China)) and cDNA was synthesized through reverse transcription with a reverse transcription kit (MH102, Yamei, Shanghai, China). Subsequently, quantitative RT-PCR was performed using SYBR Green (4913914001, Roche, Basel, Switzerland). The relative abundance of genes was determined using the 2^−ΔCt^ method. Additional file 1 contains the sequences of the qRT-PCR primers.

### NO and cytokines measurements

The supernatants were collected and used for nitric oxide (NO; Beyotime, S0021S) and ELISA measurements (DY410, R&D, Minneapolis, USA) following the guidelines provided by the manufacturer.

### Western blotting analysis

Proteins were collected with radioimmunoprecipitation assay (RIPA) lysis buffer with a cocktail of protease inhibitors (Sigma-Aldrich, P8340). Following sodium dodecyl sulfate-polyacrylamide gel electrophoresis (SDS-PAGE) electrophoresis, the proteins were moved to polyvinylidene difluoride (PVDF) membranes (ISEQ00010, Millipore, Billerica, USA). After blocking, the membranes were incubated with specific primary antibodies. The antibodies used were listed in Additional file 2.

### Fluorescence confocal microscopy analyses

For immunofluorescence analysis, PMs were treated with 4% PFA for fixation and permeabilized using 0.1% Triton X-100. Following blocking, primary antibodies were used to incubate the cells, and then with fluorescent secondary antibodies. Finally, the sections were mounted by a quench-proof tablet containing Hoechst 33342 and observed using a confocal microscope.

### Flow cytometry

Mice were intraperitoneally injected with PTX (10 μM) and PBS, respectively. On day 4, mice received an injection of 4% thioglycollate, and PMs were collected 3 days later. Cells were stained with APC-labeled anti-mouse MHC class II (#107613, Biolegend, San Diego, US) and examined using a FACSAria flow cytometer (BD Biosciences).

### Transcriptome analysis

PMs were exposed to PBS or PTX (10 μM) for 24 h, then the cells were rinsed with PBS and allowed to rest for 5 days. RNA extraction, RNA detection, quality testing, library construction, and sequencing were carried out by the Novogene company (Beijing, China). Differentially expressed genes of two conditions were performed using the edgeR R package. The clusterProfiler R package was used to perform Gene Ontology (GO) enrichment analysis and Kyoto Encyclopedia of Genes and Genomes (KEGG) functional enrichment analysis.

### Statistical analysis

Data are expressed as means ± SEM from at least three experiments. To determine statistical significance, Student’s *t*-test, one-way analysis of variance (ANOVA) with Dunnett’s multiple comparison test, or two-way ANOVA with Bonferroni’s multiple comparison test were employed. Significance was defined as *P < 0.05, **P < 0.01, ***P < 0.001, and ****P < 0.0001. GraphPad Prism6 was used for statistical analysis and graphing.

## Results

### Microtubule-stabilizing agent PTX but not microtubule-destabilizing agent Noco induces training of macrophages

**Figure 1:**
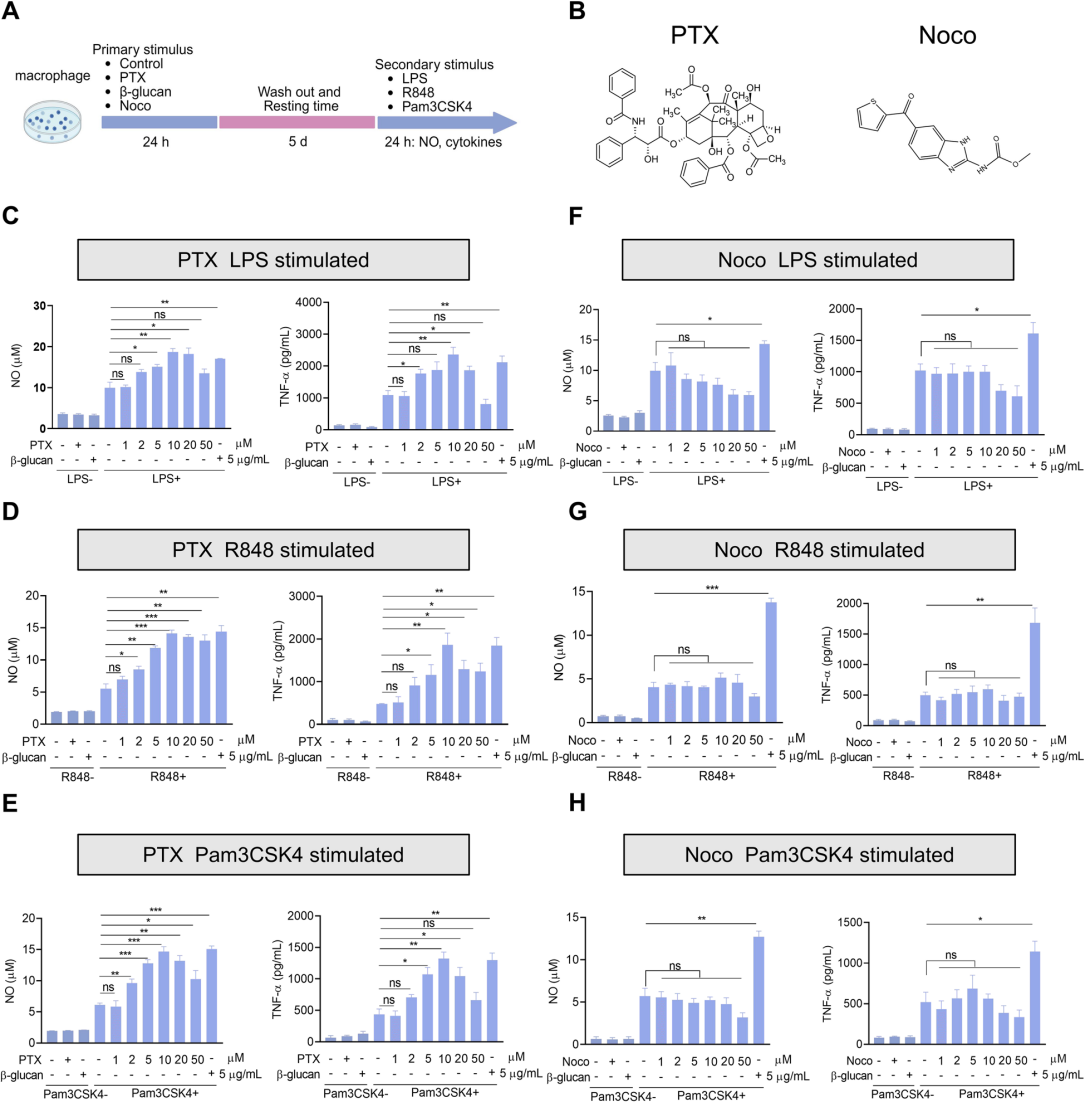
**PTX-trained macrophages exhibit enhanced expressions of inflammatory mediators after the secondary stimulus.** **A** Flow chart of in vitro trained immunity experimental procedure. **B** The chemical structural formula of PTX and Noco. PMs were pretreated with primary stimulations for 24 h and rested for 5 days. **C**–**H** Peritoneal macrophages (PMs) were restimulated with lipopolysaccharide (LPS), R848, or Pam3csk4 for 24 h, after which the production of nitric oxide (NO) and tumor necrosis factor (TNF)-a was quantified in the supernatant. Data are presented as mean ± SEM (*n* = 3). *p < 0.05, **p < 0.01, and ***p < 0.001.

Microtubule‐targeting agents(MTAs) are known to inhibit cellular proliferation and induce cytotoxicity in cancer cells, but little is known about their function in immune cells. To determine the role of MTAs in trained immunity, we trained PMs with microtubule-stabilizing agent PTX and microtubule-destabilizing agent Noco for 24 h, followed by a break of 5 days. β-glucan and PBS were utilized as positive and negative controls, respectively. And then PMs were restimulated with secondary stimulation (Figures 1A, B). PTX and Noco in the used concentration range did not affect cell viability (Additional files 3A, B). We performed functional assays by measuring the release of NO, interleukin (IL)-6, and tumor necrosis factor (TNF)-α. We found that PTX-trained PMs showed an upregulation of inflammatory mediators upon LPS restimulation, comparable to the induction observed by β-glucan training (Figure 1C and Additional file 4A), whereas Noco pretreatment did not elicit any responses compared with the control group (Figure 1F and Additional file 4B). Similarly, stimulation with the TLR7/8 agonist R848 and the TLR1/2 agonist Pam3CSK4 also showed a similar trend (Figures 1D, E, G, H). These results uncovered a novel function of PTX in inducing trained immunity.

### PTX-induced trained immunity enhances the phagocytosis and killing capacity of macrophages

**Figure 2:**
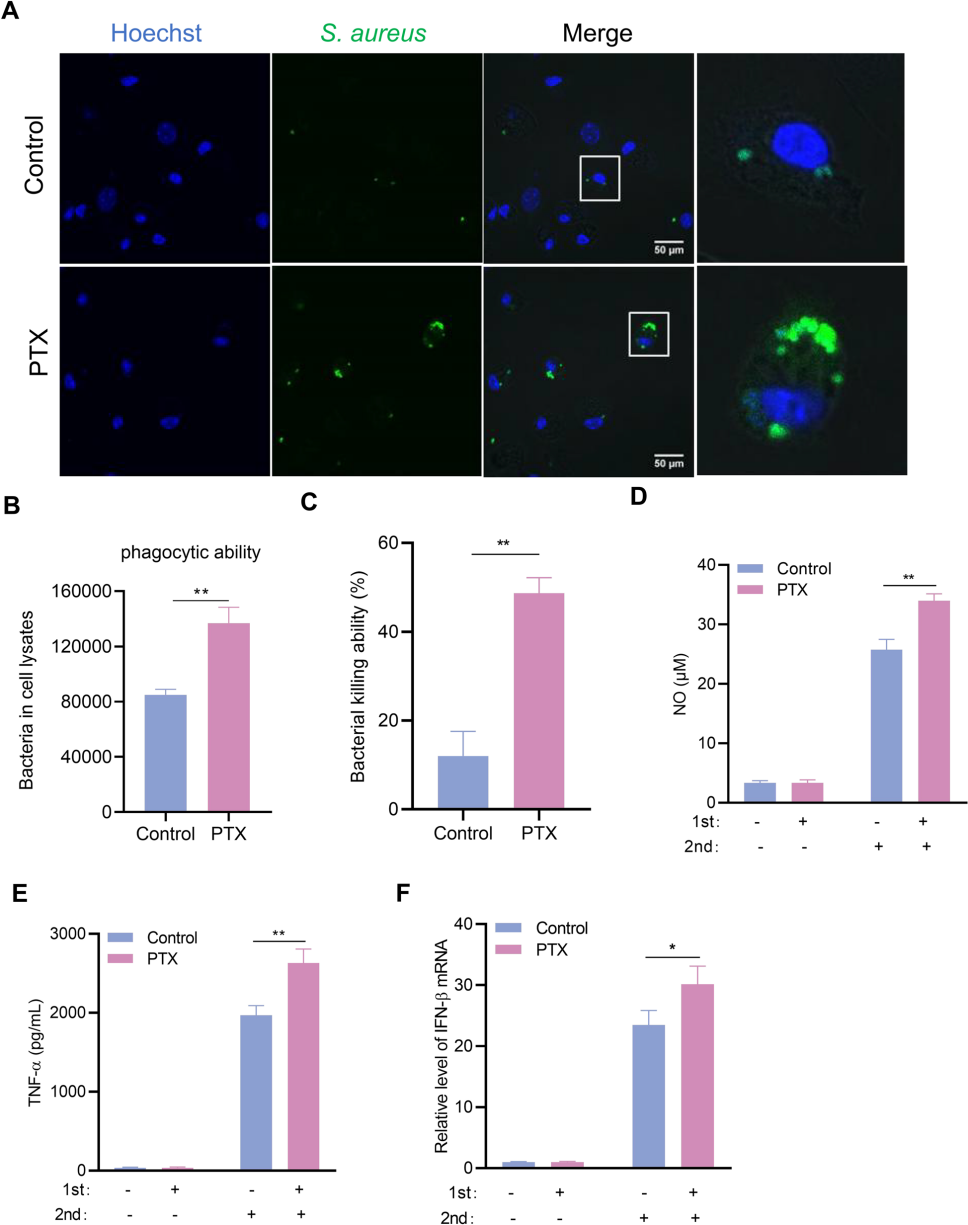
**PTX-induced trained immunity promotes macrophages to phagocytose bacteria.** PTX-trained PMs were infected with *S. aureus* (MOI = 5) for the indicated times. **A** Phagocytosis was determined by a confocal microscope. **B**, **C** Phagocytosis and killing capacity were measured by counting colony forming units (CFU). **D**, **E** NO and TNF-α production was detected in the supernatants following *S. aureus* infection. **F** interferon (IFN)-β mRNA levels were measured by qRT‑PCR. Data are presented as mean ± SEM (*n* = 3). *p < 0.05 and **p < 0.01.

Trained macrophages generally acquire a significantly enhanced functional state related to phenotypic polarization, inflammatory status, and antimicrobial activity followed by secondary stimulation. Therefore, we validated the difference in phagocytosis and killing capacity of PMs before and after PTX training. *S. aureus* was stained with FITC, and cocultured with PMs for 1 h. Green fluorescence represents intracellular phagocytosed FITC-labeled *S. aureus*. Results showed that PTX training promoted PMs to phagocytose *S. aureus* (Figures 2A, B). Moreover, we found that PTX training was sufficient to enhance bacterial killing (Figure 2C). In accordance with the above results, PTX-trained PMs presented increased levels of NO, IL-6, and TNF-α compared with the control group after infection with *S. aureus* (Figure 2D, E and Additional file 4C). These findings suggested that PTX-trained macrophages dramatically enhanced the immune response against *S. aureus* infection.

### STING pathway correlates with PTX-induced trained immunity

**Figure 3:**
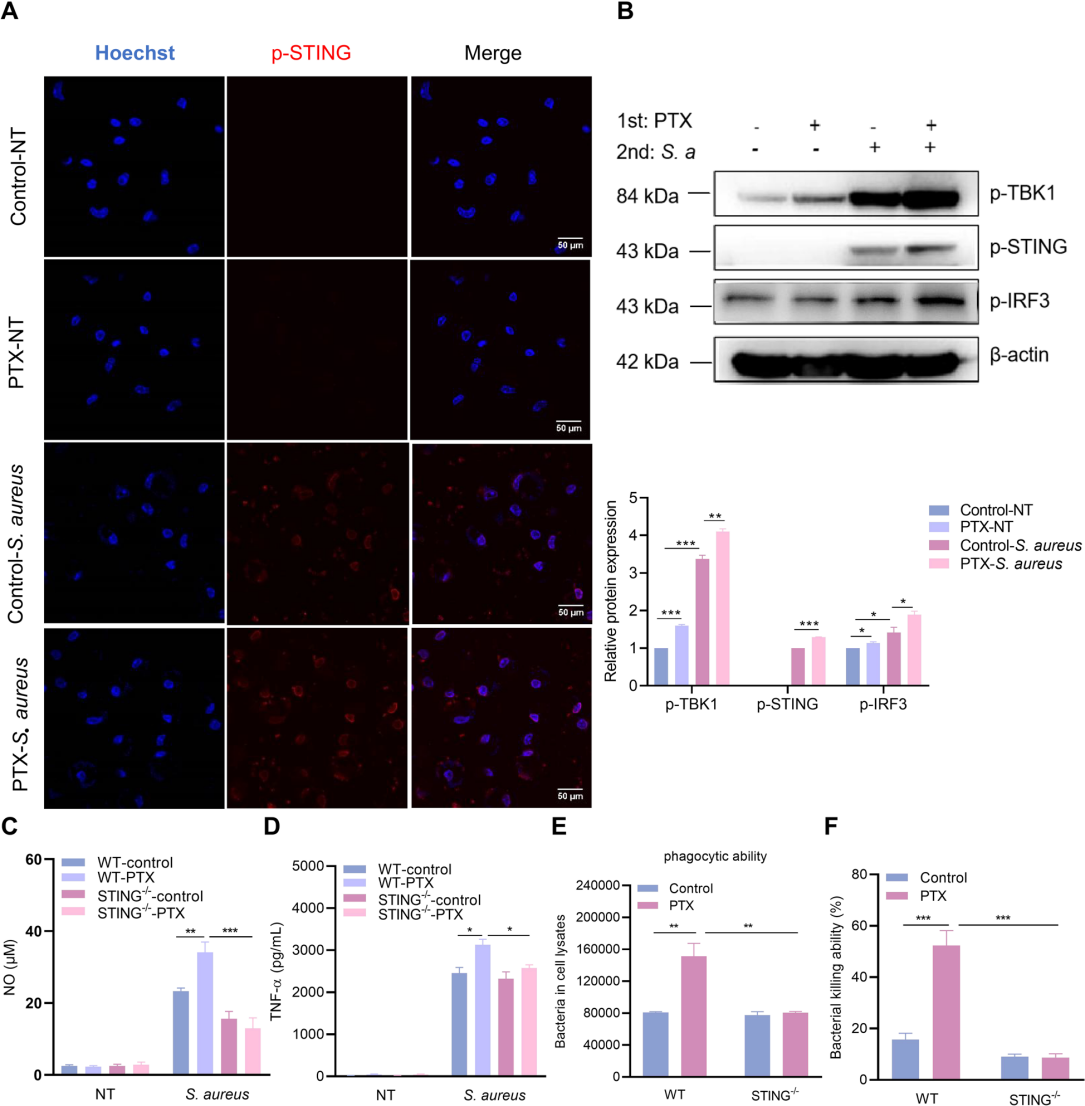
**STING pathway correlates with PTX-induced trained immunity.** PTX-trained PMs were infected with *S. aureus* (MOI = 50) for the indicated times. **A** The expression and localization of p-STING were observed by confocal microscope. **B** Western blot analysis of p-STING, p-TBK1, p-IRF3, and β-actin. **C**, **D** NO and TNF-α production was detected in the supernatants following *S. aureus* infection. **E**, **F** Phagocytosis and killing capacity were measured by counting CFUs. Data are presented as mean ± SEM (*n* = 3). *p < 0.05, **p < 0.01, and ***p < 0.001.

Combined with the above results, IFN-β levels were significantly increased in PTX-trained PMs after *S. aureus* infection (Figure 2F). Our earlier research demonstrated that the STING signaling pathway plays a crucial role in the development of HKCA-induced trained immunity [21]. To investigate the involvement of STING in PTX-induced trained immunity, we first examined the expression and subcellular localization of activated STING. The results showed that STING was phosphorylated and accumulated in the perinuclear region after *S. aureus* infection. Significantly, PTX-trained PMs showed an increase in p-STING expression as compared with the control cells (Figure 3A). Meanwhile, we found that the protein levels of p-TBK1 and p-IRF3 were upregulated in all PTX-trained cells compared with their controls (Figure 3B). Considering the robust connections between STING and PTX-trained immunity, we next investigated whether STING deficiency attenuated PTX-induced trained immunity. Wild-type (WT) and STING^−/−^ PMs were trained with PTX as described above. As expected, we found that STING remarkably affected PTX-induced training effects. STING deficiency resulted in substantially decreased levels of NO and TNF-α production after LPS stimulation following the PTX training (Figures 3C, D). Consistent with these findings, PTX-trained STING^−/−^ PMs also exhibited impaired bacterial phagocytosis and killing capacity that declined to near basal levels of WT PMs (Figures 3E, F). Overall, STING played an irreplaceable role in PTX-induced trained immunity.

### PTX triggers the switch to glycolysis in trained immunity

**Figure 4:**
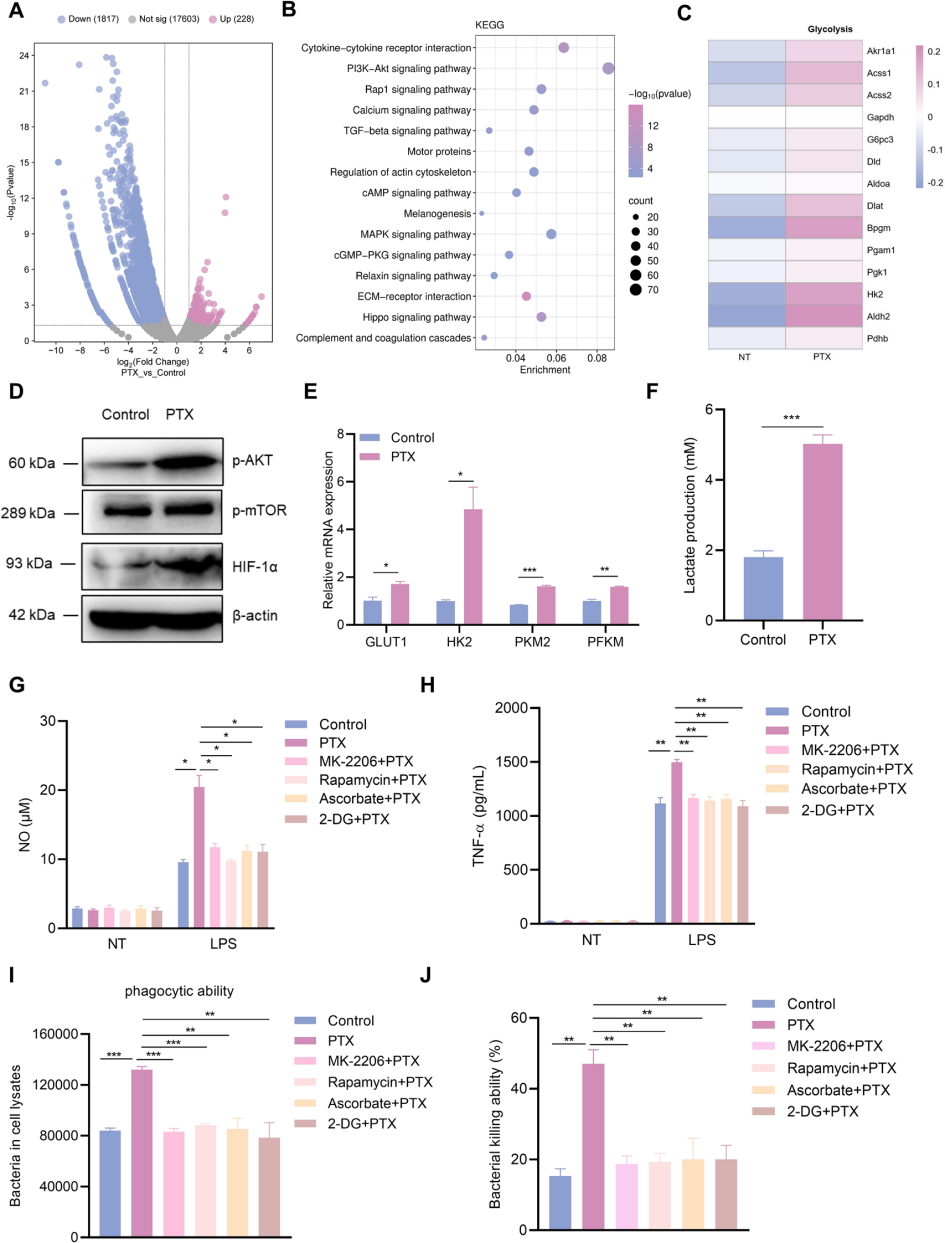
**PTX triggers the switch to glycolysis in trained immunity.** PMs were pretreated with PTX and rested for 5 days. **A** Volcano plot of differential genes. **B** Kyoto Encyclopedia of Genes and Genomes (KEGG) pathways analysis. **C** Gene expression related to glycolysis. **D** Western blot analysis of p-AKT, p-mTOR, HIF1α, and β-actin. **E** GLUT1, HK2, PKM2, and PFKM mRNA levels were measured by qRT‑PCR. **F** Lactate production was detected by an assay kit. **G**, **H** NO and TNF-α production was detected in the supernatants following LPS stimulation. **I**, **J** Phagocytosis and killing capacity were measured by counting CFUs. Data are presented as mean ± SEM (*n* = 3). *p < 0.05, **p < 0.01, and ***p < 0.001.

Epigenetic and metabolic reprogramming is fundamental in trained immunity, and both complement each other. Trained immunity is fundamentally based on the Akt–mTOR–HIF1α-driven shift from oxidative phosphorylation to aerobic glycolysis. To explore the potential mechanism by which PTX induces trained immunity, we performed a transcriptomic analysis. As shown in volcano plots, there were 2045 differentially expressed genes between control and PTX training samples, including 1817 downregulated genes and 228 upregulated genes (Figure 4A). Pathway analysis of genes with differential regulation between control and PTX-trained groups indicated the involvement of several pathways in trained immunity, such as PI3K–Akt signaling, MAPK signaling, and inflammatory cytokine-related signaling (Figure 4B). Furthermore, increased glycolysis induction was observed in PTX-trained PMs compared with that of control cells (Figure 4C). Regarding the molecular pathway, we found that PTX training led to the activation of the Akt–mTOR–HIF1α signaling pathway compared with the control cells (Figure 4D). An analysis of key glycolysis proteins was conducted, glucose transporter-1 (GLUT1), hexokinase 2 (HK2), pyruvate kinase M2 (PKM2), and phosphofructokinase (PFKM) were increased upon PTX treatment in PMs (Figure 4E). In addition, lactate production was notably higher in PTX-trained PMs than in the control group (Figure 4F). We confirmed the importance of glycolysis in the PTX training process via pathway inhibitors. The application of inhibitors resulted in the abolishment of PTX training-mediated proinflammatory responses, as well as bacterial phagocytosis and killing capacity (Figures 4G–J). These data demonstrated that PTX promoted a metabolic change from oxidative phosphorylation to aerobic glycolysis in the training process.

### PTX-induced trained immunity occurs in a GPR183–STING axis-dependent manner

**Figure 5:**
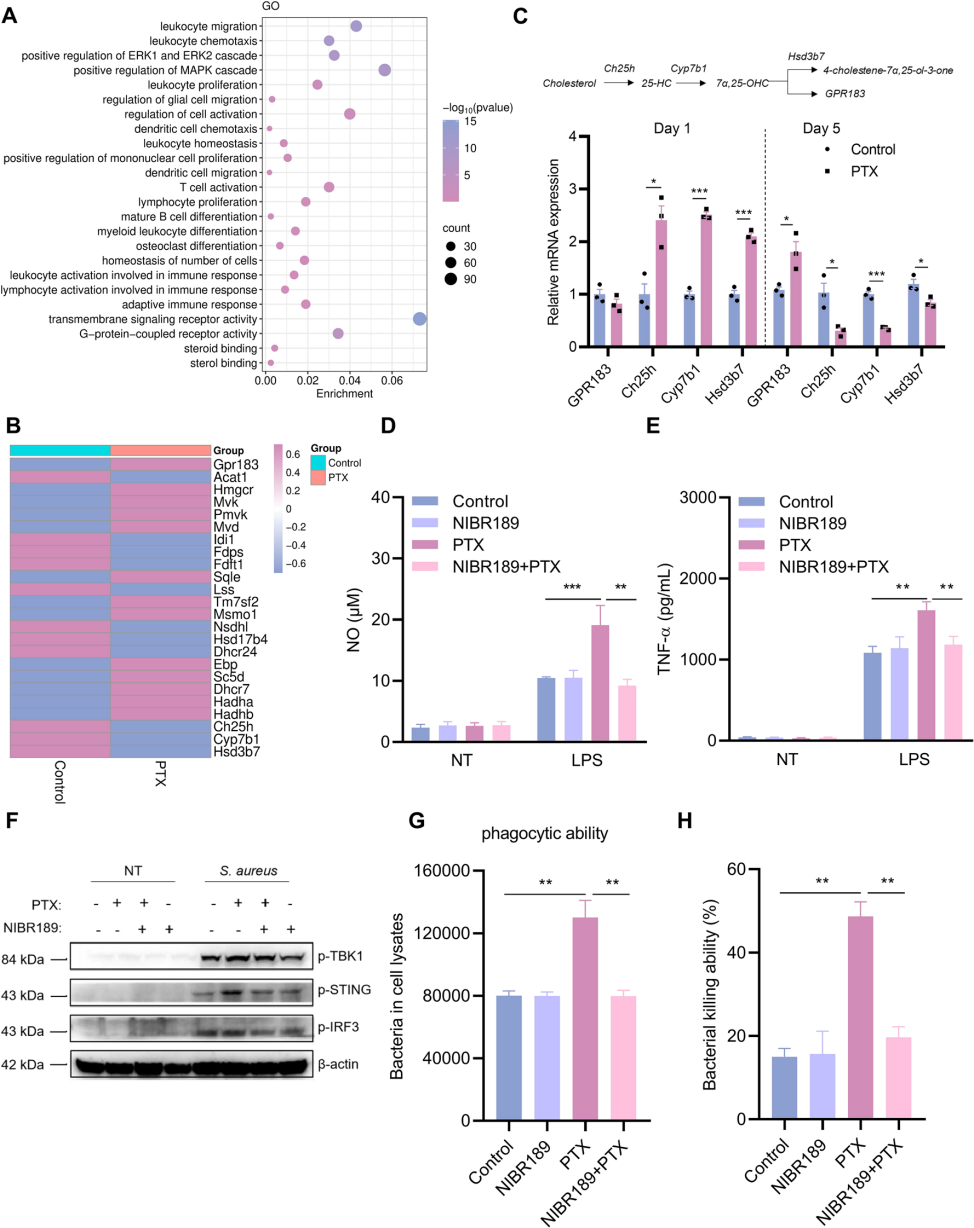
**GPR183 is the key regulator of PTX-induced trained immunity.** **A** The associated Gene Ontology (GO) enrichment analysis of the GPR183. **B** Gene expression profile associated with cholesterol metabolism. **C** GPR183, Ch25h, Cyp7b1, and Hsd3b7 mRNA levels were measured by qRT‑PCR. PMs were pretreated with NIBR189 (10 μM) for 1 h, followed by PTX training. PMs were restimulated with LPS or *S. aureus*. **D**, **E** NO and TNF-α production was detected in the supernatants following LPS stimulation. **F** Western blot analysis of p-TBK1, p-STING, p-IRF3, and β-actin. **G**, **H** Phagocytosis and killing capacity were measured by counting CFUs. Data are presented as mean ± SEM (*n* = 3). *p < 0.05, **p < 0.01, and ***p < 0.001.

To investigate how PTX induces trained immunity, we executed GO enrichment analyses. To our delight, the results manifested that GPR183 was enriched in both innate and adaptive immunity-related pathways (Figure 5A). GPR183, the receptor for potent oxysterol ligand 7α,25-dihydroxycholesterol (7α,25-OHC) and other related oxysterols, is associated with the physiological regulation of cholesterol homeostasis. The transcriptome data showed changes in the expression of the cholesterol synthesis-related genes (Figure 5B). According to the significant difference observed, we also performed qRT-PCR verification, and the results showed consistency with transcriptome results. We found that the 7α,25-OHC synthesis genes Ch25h, Cyp7b1, and decomposition enzyme Hsd3b7 were all expressed at higher levels on the first day after PTX training. On the fifth day, the expressions of Ch25h, Cyp7b1, and Hsd3b7 were all significantly downregulated, suggesting an autoregulatory feedback mechanism for 7α,25-OHC production, eventually leading to the enhanced or sustained activation of GPR183 (Figure 5C). Subsequently, NIBR189, a specific GPR183 inhibitor, was used for further validation. When PMs were pretreated with NIBR189, PTX training-induced proinflammatory mediator (NO and TNF-α) production was significantly inhibited upon LPS restimulation (Figures 5D, E). Consistently, after PTX training, the expressions of STING-related protein were even more inhibited following treatment with NIBR189 upon *S. aureus* infection (Figure 5F). Consistent with the above results, the addition of NIBR189 reduced the phagocytosis and killing capacity (Figures 5G, H). Overall, the data indicated that PTX-induced trained immunity relied on the GPR183–STING axis.

### PTX-induced trained immunity enhances host protection against *S. aureus* pneumonia via the GPR183–STING axis

**Figure 6:**
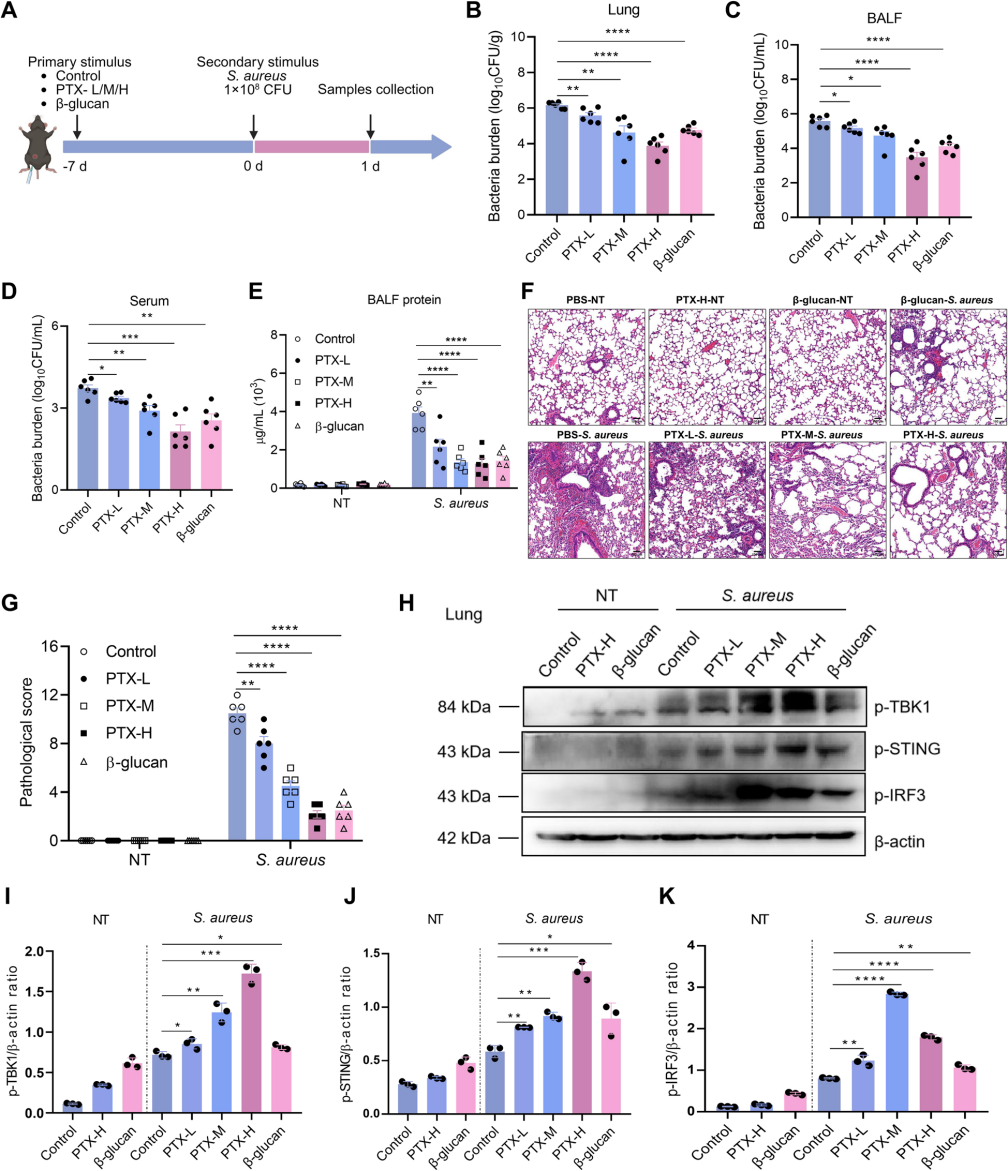
**PTX-induced trained immunity prevents mice against** ***S. aureus*** **pneumonia**. Mice received a single intraperitoneally injection of PTX-L, PTX-M, PTX-H, β-glucan, or PBS, and 7 days thereafter, mice were infected intranasally with S. aureus. **A** Flow chart of in vivo trained immunity experimental procedure. **B**–**D** The bacterial burden in serum, BALF, and lung tissue homogenates was determined. **E** Total protein in BALF was measured. **F** Lung tissue structures were observed by H&E staining. **G** Pathological scores of lung tissues. **H**–**K** Western blot analysis of p-TBK1, p-STING, p-IRF3, and β-actin. Data are presented as mean ± SEM (*n* = 6). *p < 0.05, **p < 0.01, ***p < 0.001, and ****p < 0.0001.

PTX is a well-known chemotherapeutic agent in cancer therapy. Our study reveals a novel function for PTX as a strong inducer of trained immunity. To study in vivo relevance, we established the pneumonia model by intranasally injecting *S. aureus* to evaluate the antibacterial ability of PTX-induced trained immunity in vivo (Figure 6A). In comparison to the control, PTX-trained mice exhibited dose-dependently decreased bacterial burden in the serum, lung, and BALF at 24 h post-infection (Figures 6B–D). Lung barrier function, as evaluated by measurements of total protein content in BALF, was markedly disrupted after *S. aureus* challenge, and PTX training significantly reversed the protein leak in BALF (Figure 6E). Histological analysis of lung tissue showed pulmonary interstitial edema, infiltration of inflammatory cells, rupture of pulmonary alveoli, and hemorrhage in the control group, whereas PTX training significantly alleviated symptoms after *S. aureus* infection (Figures 6F, G). Consistent with these results, the levels of STING-related protein were significantly activated in the PTX training group (Figure 6H). To examine the effectiveness of PTX-induced trained immunity in vivo, mice were pretreated with PTX, macrophages were subsequently harvested and subjected to activity analysis (Additional file 5A). We found that PTX-trained macrophages exhibited higher expression of MHC class II, heightened production of NO and TNF-α, and enhanced bacterial phagocytosis and killing capacity compared with the control group (Additional files 5B–F). Overall, these observations suggested that PTX-induced trained immunity enhanced host resistance to *S. aureus* pneumonia.

**Figure 7:**
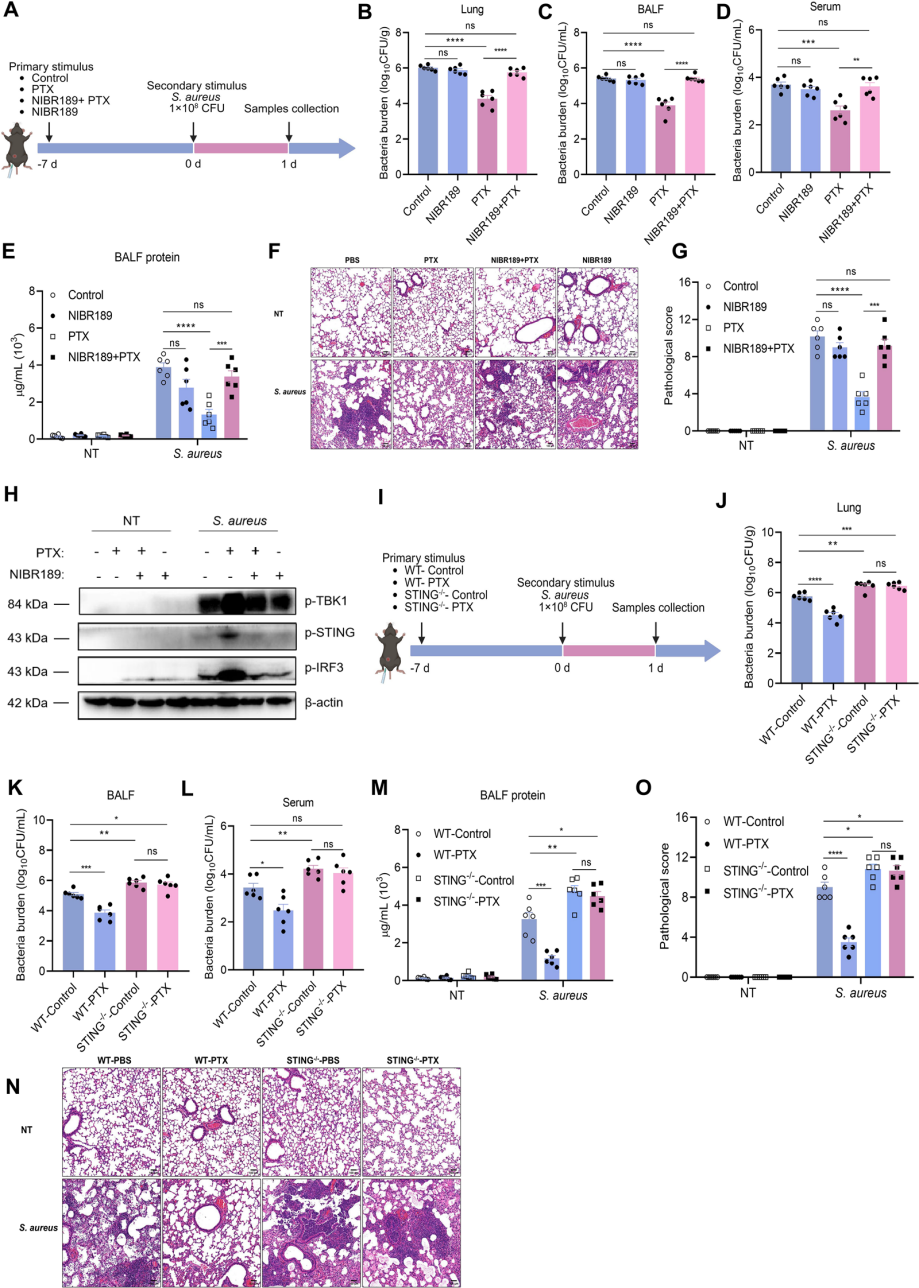
**Blocking the GPR183–STING axis in PTX-trained mice impairs host defense against** ***S. aureus*** **pneumonia.** **A** Flow chart of in vivo trained immunity experimental procedure. **B**–**D** The bacterial burden in serum, BALF, and lung tissue homogenates was determined. **E** Total protein in BALF was measured. **F** Lung tissue structures were observed by H&E staining. **G** Pathological scores of lung tissues. **H** Western blot analysis of p-TBK1, p-STING, p-IRF3, and β-actin. WT and STING^−/−^ mice received a single intraperitoneally injection of PTX or PBS and 7 days thereafter, mice were infected intranasally with *S. aureus*. **I** Flow chart of in vivo trained immunity experimental procedure. **J**–**L** The bacterial burden in serum, BALF, and lung tissue homogenates was determined. **M** Total protein in BALF was measured. **N** Lung tissue structures were observed by H&E staining. **O** Pathological scores of lung tissues. Data are presented as mean ± SEM (*n* = 6). *p < 0.05, **p < 0.01, ***p < 0.001, and ****p < 0.0001.

We then assessed if blocking the GPR183–STING pathway in mice trained with PTX would weaken their defense against *S. aureus* pneumonia. The mice received an intraperitoneal injection of NIBR189 or a vehicle 1 h before PTX training (Figure 7A). Consistent with the in vitro results, the PTX-trained mice treated with NIBR189 exhibited increased protein leak in BALF, more severe pathological damage to lung tissue, and enhanced bacterial burden in the serum, lung, and BALF, comparable to the results found in the control group of mice (Figures 7B–G). Moreover, NIBR189 treatment resulted in decreased expressions of STING-related proteins (Figure 7H). There was no notable alteration in bacterial load, protein leakage, and tissue damage in STING^−/−^ mice before and after PTX training (Figures 7I–O). Collectively, these findings indicated that the GPR183–STING axis played a key role in host defense against *S. aureus* pneumonia during PTX training.

## Discussion

The global emergence of MRSA represents a formidable challenge to public health, necessitating novel preventive and therapeutic strategies. Trained immunity has emerged as a promising approach to enhancing host defense against MRSA infections. In response to the growing restrictions on antibiotic use, plant-derived natural compounds have garnered attention as potential alternatives for combating MRSA. While PTX is primarily known as a chemotherapeutic agent, its antiinflammatory, antifibrotic, and neuroprotective properties suggest broader applications in infection control, immune regulation, and neuroregeneration. This investigation revealed that PTX-induced trained immunity through the GPR183–STING axis, a pathway crucial for enhancing host resistance against MRSA pneumonia. These findings highlight the potential of PTX as an immunomodulatory agent and provide new insights into its role in infection control.

As typical representatives of innate immunity, trained macrophages are generally equipped with enhanced proinflammatory and phagocytic abilities [24]. In this research, we initially showed that PTX-trained macrophages produced more inflammatory mediators after multiple secondary stimulations, but there was no notable difference between the Noco and PBS groups. Besides inflammatory mediators, phagocytosis by macrophages is vital for preserving cellular homeostasis and the host cell defense system. Our results showed that PTX treatment boosted phagocytosis and the bacterial killing ability response to *S. aureus* infection, accompanied by excessive production of the proinflammatory mediators NO and TNF-α. These observations indicated that PTX effectively programed macrophages into a more active and responsive state, reinforcing their protective role in infection control.

Trained immunity involves enhanced expression of germline-encoded pattern-recognition receptors (PRRs), which are deeply relevant to the memory-like feature. Numerous studies have confirmed the importance of the NOD-like receptors, the Toll-like receptors, and the C-type lectin receptors in trained immunity [25–28]. Our findings and previous studies shine a light on the strong involvement of the STING signaling pathway in trained immunity [21, 29]. In this study, PTX-induced trained immunity activated STING signaling and the enhanced inflammatory responses disappeared after STING deficiency. These observations indicated that STING signaling activation may serve as a feature of the trained immunity functional phenotype. Previous studies reported that STING drives HIF-1α stabilization and metabolic reprogramming, which subsequently functions to defend against *Brucella* infection [30]. Bacillus Calmette–Guérin (BCG) with increased c-di-AMP expression exhibits more pronounced characteristics of trained immunity including stronger immune response and enhanced epigenetic and metabolomic changes [29]. These studies may provide referable insights into deep action mechanisms between STING and trained immunity.

Trained immunity is accompanied by epigenetic and metabolic reprogramming. Characteristically, trained cells frequently reprogram their own metabolic pathways from oxidative phosphorylation to aerobic glycolysis [31]. The common denominator in this process appears to be the Akt–mTOR–HIF1α pathway and the TCA cycle, and their interaction regulates histone modifications thereby influencing gene expression [7]. In this context, we found that PTX training could induce metabolic reprogramming with enhanced levels of Akt–mTOR–HIF1α proteins, glycolytic enzymes, and lactate production. Similar to BCG- and oxLDL-trained monocytes, the PTX-trained macrophages were also associated with enhanced glycolysis [32, 33]. Pharmacological inhibition of the Akt–mTOR–HIF1α pathway resulted in the impairment of PTX-induced trained immunity. To further explore the regulatory mechanism through which PTX regulates trained immunity, we carried out transcriptome analysis. Intriguingly, GPR183 was significantly increased after PTX training. Inhibiting the GPR183 with NIBR189 weakened macrophage functions, as evidenced by decreased proinflammatory and antimicrobial immune responses upon LPS or *S. aureus* challenge. Notably, NIBR189-pretreatment macrophages were also accompanied by decreased levels of the STING-associated proteins after *S. aureus* infection, suggesting a crucial link between GPR183 and STING signaling in PTX-induced trained immunity. Earlier research has indicated that GPR183 is crucial for metabolism and functions in both innate and adaptive immune cells, linking it to various inflammatory and autoimmune diseases [34]. Our findings uncover a novel function of GPR183 and suggest a new mechanism for regulating trained immunity.

The trained immunity-mediated enhanced functional state is a double-edged sword that depends on the differential pathological status [35]. Trained immunity could provide robust innate immunity against future threatening attackers (e.g., infections and cancer) [36, 37]. However, the persistence of trained immunity can promote the development of various diseases (e.g., autoimmunity and transplantation) [38, 39]. Interestingly, trained immunity can also reverse innate immune tolerance [40]. PTX has long been a cornerstone of cancer therapy, yet its effects on the innate immune system remain largely unexplored. This gap in knowledge may lead to uncertainties in clinical medication and treatment guidance. In the present study, we demonstrated that PTX training significantly enhanced host resistance to MRSA pneumonia, further supporting its potential as an immunomodulatory agent. These findings provide new insights into PTX’s broader biological functions and highlight its potential for repurposing in infection control and immune regulation.

## Supplementary Information

**Additional file 1**. **Sequences of forward and reverse primers used for PCR amplification.**

**Additional file 2**. **Antibody used for western blot and immunofluorescence**.

**Additional file 3**. **Effects of PTX and Noco on the viability in macrophages.** (A, B) Cell growth was determined by CCK8 assay. Data are presented as mean ± SEM (*n* = 3).

**Additional file 4**. **Detection of the levels of IL-6.** PMs were pretreated with primary stimulations for 24 h and rested for 5 d. (A–C) PMs were restimulated with LPS, R848, Pam3csk4, or *S. aureus* for 24 h, after which the production of IL-6 was quantified in the supernatant. Data are presented as mean ± SEM (*n* = 3). * *p* < 0.05, ** *p* < 0.01, and *** *p* < 0.001.

**Additional file 5**. **Macrophages derived from PTX-trained mice exhibit enhanced functional state after the secondary stimulus.** (A) Experimental scheme. (B) The expression of MHC class II on macrophages at 7 days post-PTX exposure. (C, D) NO and TNF-α production was detected in the supernatants following LPS stimulation. (E, F) Phagocytosis and killing capacity were measured by counting CFU. Data are presented as mean ± SEM (*n* = 3). * *p* < 0.05, ** *p* < 0.01, and *** *p* < 0.001.

## Data Availability

No datasets were generated or analysed during the current study.

## Abbreviations

AMR: antimicrobial resistance
BALF: bronchoalveolar lavage fluid
BPPL: bacterial priority pathogens list
CA-MRSA: community-acquired MRSA
GLUT1: glucose transporter-1
HA-MRSA: healthcare-acquired MRSA
HK2: hexokinase 2
H&E: hematoxylin and eosin
HKCA: heat-killed *Candida albicans*
LA-MRSA: livestock-associated MRSA
MOI: multiplicity of infection
MRSA: methicillin-resistant *Staphylococcus aureus*
MTAs: microtubule‐targeting agents
NO: nitric oxide
PBS: phosphate-buffered saline
PFKM: phosphofructokinase
PKM2: pyruvate kinase M2
PMs: peritoneal macrophages
PRRs: pattern-recognition receptors
PTX: paclitaxel
SSIs: surgical site infections
WT: wild-type
7α,25-OHC: 7α,25-dihydroxycholesterol
