# Supplementary material for: Paclitaxel induces trained immunity via the GPR183–STING axis to enhance host defense against MRSA infection
Source: Vet Res. 2026 Jan 16;57:30. doi: 10.1186/s13567-025-01704-8 (PMC12892545; doi:10.1186/s13567-025-01704-8)
Supplement: Supplementary file 1 — Additional file 1. Sequences of forward and reverse primers used for PCR amplification. [file 13567_2025_1704_MOESM1_ESM.docx]

Addition file 1. Sequences of forward and reverse primers used for PCR amplification

| Gene | Forward Primer | Reverse Primer |
| --- | --- | --- |
| β-actin | CGTGGGCCGCCCTAGGCACCA | TTGGCCTTAGGGTTCAGGGGGG |
| IFN-β | ACTGCCTTTGCCATCCAAGA | CACTGTCTGCTGGTGGAGTT |
| GLUT1 | CAGTTCGGCTATAACACTGGTG | GCCCCCGACAGAGAAGATG |
| HK2 | TGATCGCCTGCTTATTCACGG | AACCGCCTAGAAATCTCCAGA |
| PKM2 | GCCGCCTGGACATTGACTC | CCATGAGAGAAATTCAGCCGAG |
| PFKM | TGTGGTCCGAGTTGGTATCTT | GCACTTCCAATCACTGTGCC |
| GPR183 | GTCGTGTTCATCCTGTGCTTCAC | TCATCAGGCACACCGTGAAGTG |
| Ch25h | CTGACCTTCTTCGACGTGCT | GGGAAGTCATAGCCCGAGTG |
| Cyp7b1 | CGGAAATCTTCGATGCTCCAAAG | GCTTGTTCCGAGTCCAAAAGGC |
| Hsd3b7 | ACTGCGCTTTGGAGGTCGTCTA | GCCACCAGTATGTGCATCCAAG |
