# Supplementary material for: Paclitaxel induces trained immunity via the GPR183–STING axis to enhance host defense against MRSA infection
Source: Vet Res. 2026 Jan 16;57:30. doi: 10.1186/s13567-025-01704-8 (PMC12892545; doi:10.1186/s13567-025-01704-8)
Supplement: Supplementary file 2 — Additional file 2. Antibody used for western blot and immunofluorescence. [file 13567_2025_1704_MOESM2_ESM.docx]

Addition file 2. Antibody used for western blot and immunofluorescence

| Protein | Brand | Cat.NO. |
| --- | --- | --- |
| p-mTOR | Abcam | ab109268 |
| p-AKT | Proteintech | 66444-1-Ig |
| HIF1α | Abcam | ab179483 |
| p-STING | Cell Signaling Technology | 72971 |
| p-TBK1 | Cell Signaling Technology | 5483 |
| p-IRF3 | Cell Signaling Technology | 29047 |
| β-actin | Proteintech | 81115-1-RR |
| β-Tubulin | Yeasen | 30301ES |
