# Supplementary material for: Paclitaxel induces trained immunity via the GPR183–STING axis to enhance host defense against MRSA infection
Source: Vet Res. 2026 Jan 16;57:30. doi: 10.1186/s13567-025-01704-8 (PMC12892545; doi:10.1186/s13567-025-01704-8)
Supplement: Supplementary file 3 — Additional file 3. Effects of PTX and Noco on the viability in macrophages. (A, B) Cell growth was determined by CCK8 assay. Data are presented as mean ± SEM (n = 3). [file 13567_2025_1704_MOESM3_ESM.docx]

**
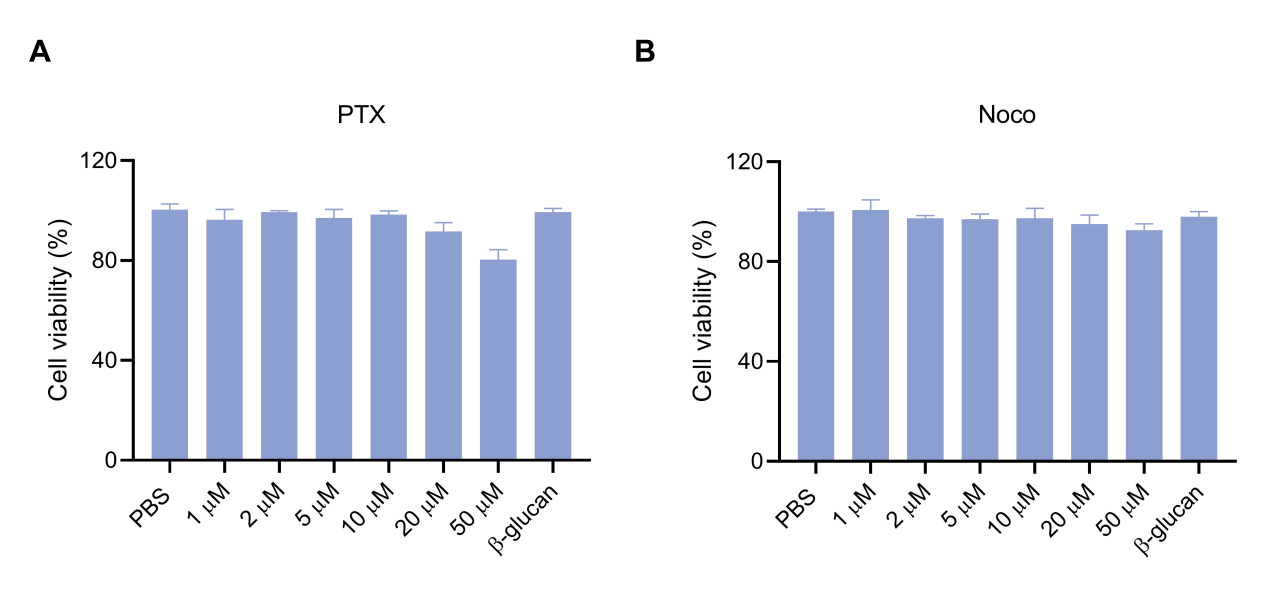
**

**Addition file 3 Effects of PTX and Noco on the viability in macrophages** (A, B) Cell growth was determined by CCK8 assay. Data are presented as mean ± SEM (n=3).
