# Supplementary material for: Paclitaxel induces trained immunity via the GPR183–STING axis to enhance host defense against MRSA infection
Source: Vet Res. 2026 Jan 16;57:30. doi: 10.1186/s13567-025-01704-8 (PMC12892545; doi:10.1186/s13567-025-01704-8)
Supplement: Supplementary file 4 — Additional file 4. Detection of the levels of IL-6. PMs were pretreated with primary stimulations for 24 h and rested for 5 d. (A–C) PMs were restimulated with LPS, R848, Pam3csk4, or S. aureus for 24 h, after which the production of IL-6 was quantified in the supernatant. Data are presented as mean ± SEM (n = 3). * p < 0.05, ** p < 0.01, and *** p < 0.001. [file 13567_2025_1704_MOESM4_ESM.docx]

**
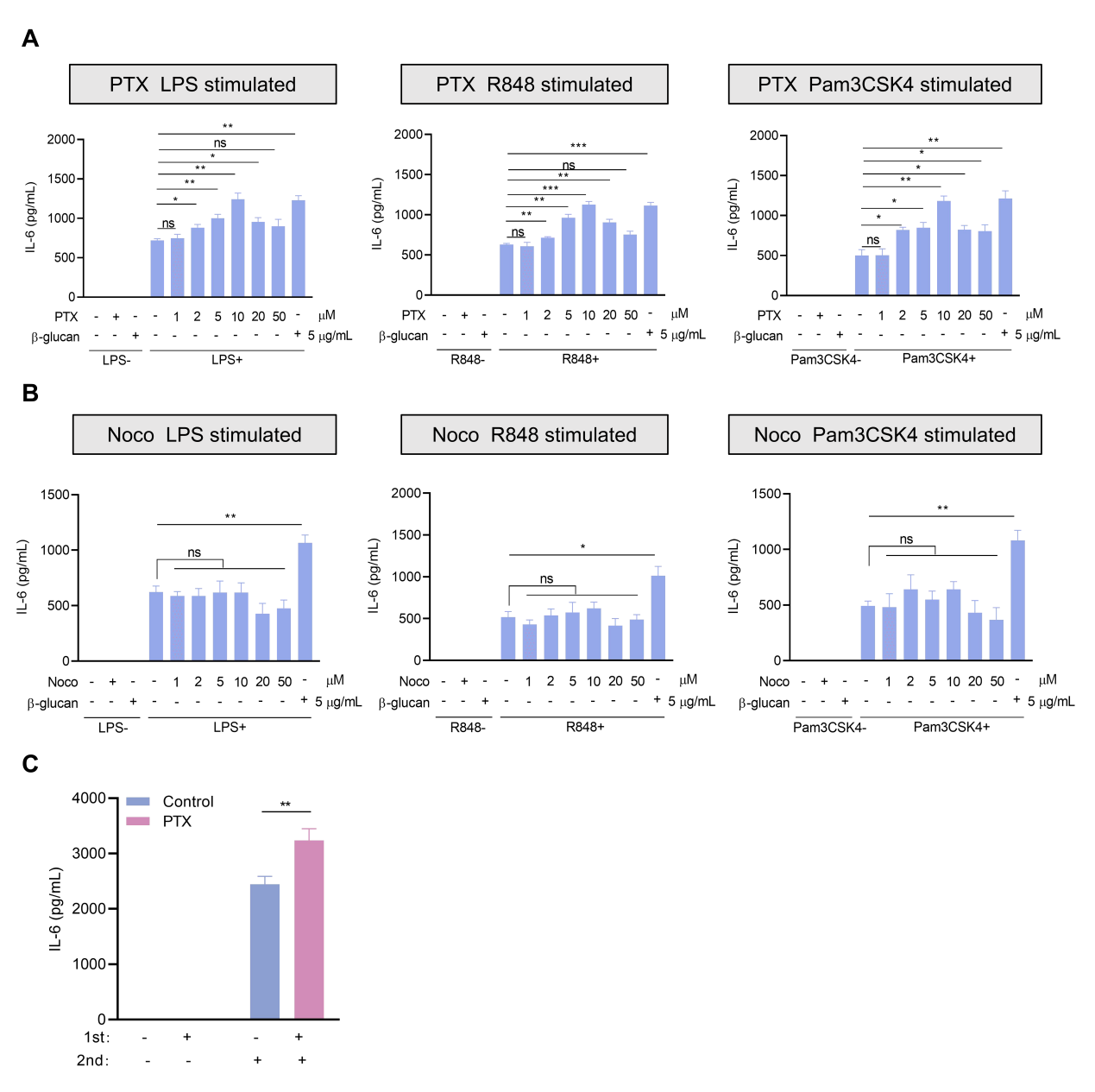
**

**Addition file 4 Detection of the levels of IL-6.** PMs were pretreated with primary stimulations for 24 h and rested for 5 d. (A-C) PMs were restimulated with LPS, R848, Pam3csk4, or *S. aureus* for 24 h, after which the production of IL-6 was quantified in the supernatant. Data are presented as mean ± SEM (n=3). * p < 0.05, ** p < 0.01, and *** p < 0.001.
