# Supplementary material for: Paclitaxel induces trained immunity via the GPR183–STING axis to enhance host defense against MRSA infection
Source: Vet Res. 2026 Jan 16;57:30. doi: 10.1186/s13567-025-01704-8 (PMC12892545; doi:10.1186/s13567-025-01704-8)
Supplement: Supplementary file 5 — Additional file 5. Macrophages derived from PTX-trained mice exhibit enhanced functional state after the secondary stimulus. (A) Experimental scheme. (B) The expression of MHC class II on macrophages at 7 days post-PTX exposure. (C, D) NO and TNF-α production was detected in the supernatants following LPS stimulation. (E, F) Phagocytosis and killing capacity were measured by counting CFU. Data are presented as mean ± SEM (n = 3). * p < 0.05, ** p < 0.01, and *** p < 0.001. [file 13567_2025_1704_MOESM5_ESM.docx]

**
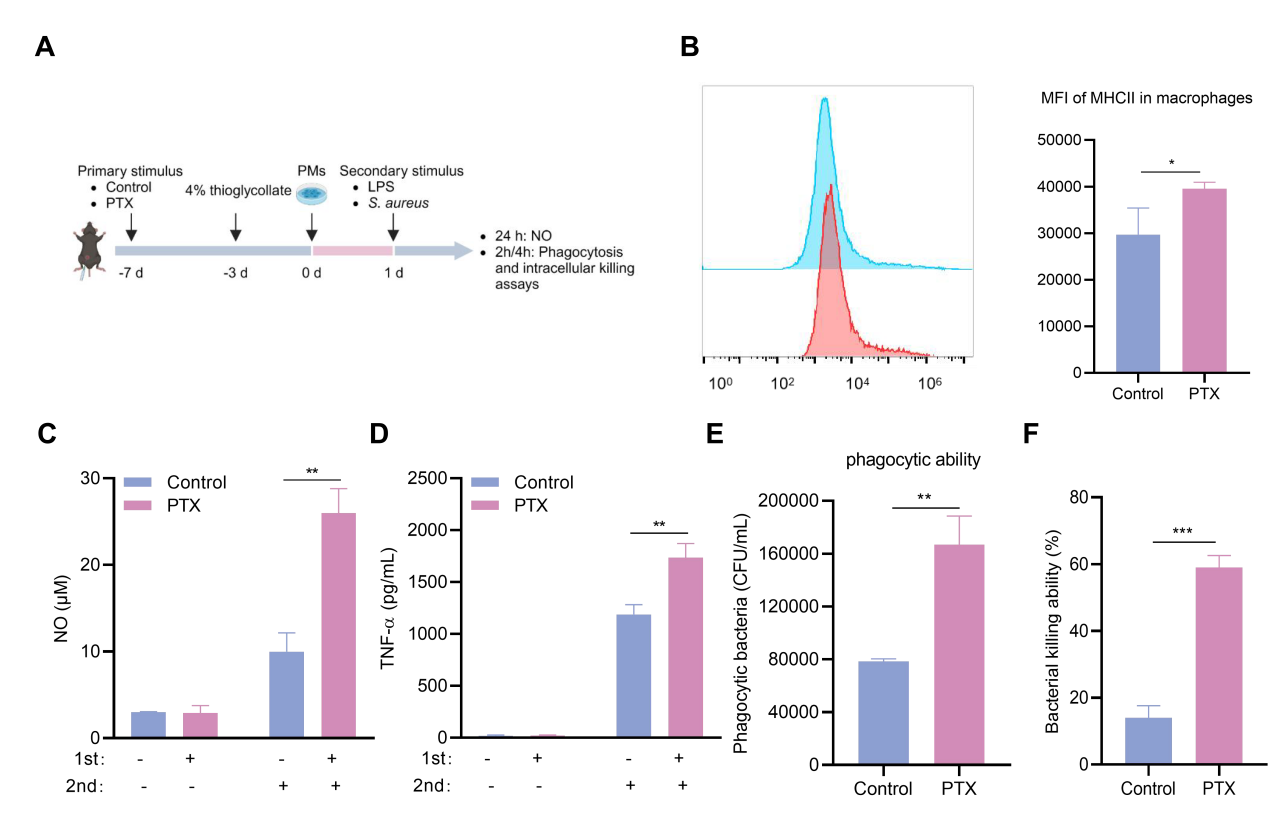
**

**Addition file 5 Macrophages derived from PTX-trained mice exhibit enhanced functional state after the secondary stimulus.** (A) Experimental scheme. (B) The expression of MHC class II on macrophages at 7 days post-PTX exposure. (C, D) NO and TNF-α production was detected in the supernatants following LPS stimulation. (E, F) Phagocytosis and killing capacity were measured by counting CFU. Data are presented as mean ± SEM (n=3). * p < 0.05, ** p < 0.01, and *** p < 0.001.
